# Fat‐Free Mass: Friend or Foe to Metabolic Health?

**DOI:** 10.1002/jcsm.13714

**Published:** 2025-02-02

**Authors:** Christopher J. Oliver, Mike Climstein, Nedeljka Rosic, Anja Bosy‐Westphal, Grant Tinsley, Stephen Myers

**Affiliations:** ^1^ Faculty of Health Southern Cross University Lismore NSW Australia; ^2^ Clinical and Health Services Faculty of Health Southern Cross University Bilinga QLD Australia; ^3^ Exercise and Sport Science Exercise, Health & Performance Faculty Research Group Faculty of Health Sciences University of Sydney Sydney NSW Australia; ^4^ Faculty of Health Southern Cross University Bilinga QLD Australia; ^5^ Institut für Humanernährung und Lebensmittelkunde Christian‐Albrechts‐Universität zu Kiel Kiel Germany; ^6^ Department of Kinesiology & Sport Management Texas Tech University Lubbock Texas USA; ^7^ NatMed‐Research Evans Head NSW Australia

**Keywords:** ectopic fat, fat‐free mass index, metabolic health, resistance training, skeletal muscle

## Abstract

**Background:**

Fat mass (FM) and fat‐free mass (FFM) are body composition estimates commonly reported in research studies and clinical settings. Recently, fat‐free mass indexed to height (fat‐free mass index; FFMI) has been shown to be positively associated with impaired insulin sensitivity or insulin resistance. Consequently, hypertrophic resistance training which can increase FFM was also questioned. This paper sets out to evaluate these propositions.

**Methods:**

In this narrative review, we discuss possible reasons that link FFMI to adverse metabolic health outcomes including the limitations of the body composition model that utilizes FFM. The safety of resistance training is also briefly discussed.

**Results:**

Approximately 50% of FFM is comprised of skeletal muscle (SM), with the other 50% being viscera, skin, and bone; FFM and SM cannot be conflated. FFM and fat mass (FM) can both rise with increasing body weight and adiposity, indicating a positive correlation between the two compartments. Risk assessment models not adequately adjusting for this correlation may cause erroneous conclusions, however which way FM and FFM are indexed. Adipose tissue accumulation with weight gain, measured by dual‐energy X‐ray absorptiometry or bioelectrical impedance, can inflate FFM estimates owing to increased connective tissue. Increased adiposity can also result in fat deposition within skeletal muscle disrupting metabolic health. Importantly, non‐skeletal muscle components of the FFM, i.e., the liver and pancreas, both critical in metabolic health, can also be negatively affected by the same lifestyle factors that impact SM. The most frequently used body composition techniques used to estimate FM and FFM cannot detect muscle, liver or pancreas fat infiltration. Prospective evidence demonstrates that resistance training is a safe and effective exercise modality across all ages, especially in older adults experiencing age‐ or disease‐related declines in muscle health.

**Conclusions:**

The association between FFM and insulin resistance is largely an artefact driven by inadequate assessment of skeletal muscle. If FM and FFM are used, at the minimum, they need to be evaluated in context with one another. Body composition methods, such as magnetic resonance imaging, which measures skeletal muscle rather than fat‐free mass, and adipose tissue as well as muscle ectopic fat, are preferred methods. Resistance training is important in achieving and maintaining good health across the lifespan. While strength and power are critical components of resistance training, the reduction of skeletal mass through ageing or disease may require hypertrophic training to mitigate and slow down the progression of this often‐inevitable process.

## 1 Introduction

In a number of human studies, fat‐free mass (FFM) has been negatively linked to metabolic health [1, 2, 3, 4]. The premise underpinning this negative association is that the fat‐free mass index (FFMI) which is FFM indexed to height (FFM/Ht^2^) is positively associated with impaired insulin sensitivity or insulin resistance. This means the higher the FFM, the worse the insulin resistance. This association between FFM and insulin resistance has been outlined in a systematic review by Perreault et al. [5] and in more recent papers [1, 3, 4, 6]. It was argued that studies in which FFM expressed, for example as a percentage of body mass, have shown a detrimental effect of lower FFM on metabolic health are considered erroneous, as a low FFM often represents a high fat mass (FM) [5]. The negative association between the FFMI and metabolic health has also led to concerns about hypertrophic resistance training potentially having detrimental effects on metabolic health [7].

Several explanations were offered by Lagace et al. to explain the association between a higher FFMI and insulin resistance or metabolic syndrome [2]; 1) the infiltration of lipids into skeletal muscle, 2) a greater FFM is usually characterized by higher proportions of type 2 fibres compared with type 1 fibres, and 3) a reduced capillary‐to‐muscle fibre ratio and decreased capillary density in individuals with a higher FFM. Central to the thesis of this paper, is that the three reasons given for this association pertain directly to skeletal muscle and not necessarily to fat‐free mass. We argue that a major cause of the FFMI‐metabolic health association can be found in the fundamental limitations of the two‐compartment body composition (2‐C bc) model, which are used to measure FFM; with the limitations becoming more apparent with increasing adiposity and obesity. In the paper, we present our argument by outlining the limitations of the 2‐C bc model and discussing two other critical factors (poor diet and inadequate physical activity) that negatively affect both adipose tissue and skeletal muscle and are the most likely key drivers behind insulin resistance.

### 1.1 Two Compartment Model

**FIGURE 1:**
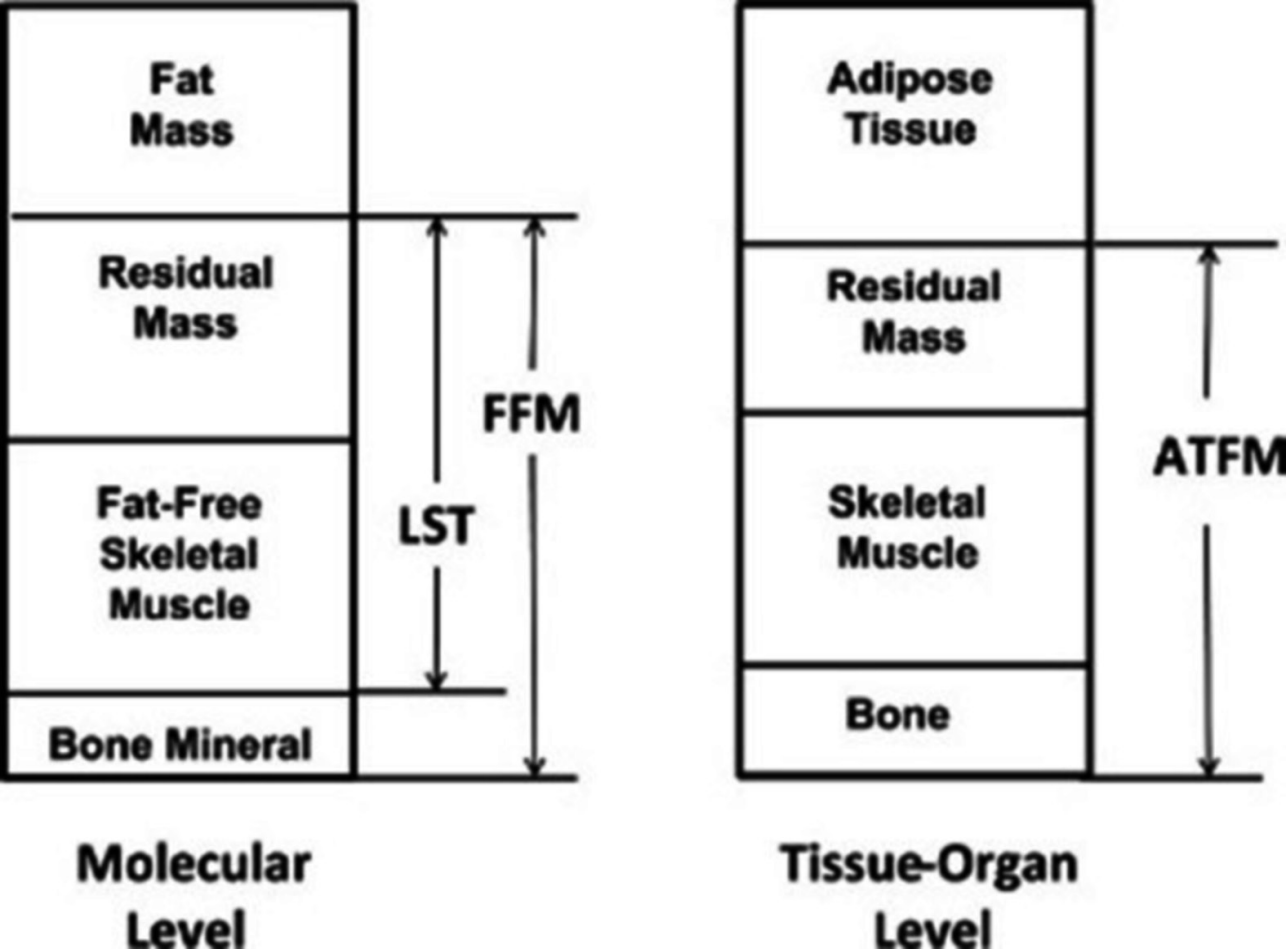
Selected molecular and tissue‐organ body composition level components. ATFM, adipose‐tissue free mass; FFM, fat‐free mass; LST, lean soft tissue. Residual mass includes organs such as liver and pancreas. Used with permission [8].

Fat‐free mass, along with fat mass are widely used parameters in clinical, body composition, and sport research, as well as in healthcare settings. The two‐compartment body composition model is expressed as body mass (BM) = FM + FFM. Fat mass, like FFM, can also be indexed to height, i.e., FM/Ht^2^ (FFMI). Another similar term to FFM is lean soft tissue, which is FFM minus the bone mineral content, Figure 1. At the molecular level, FFM consists of all non‐fat molecules in the body, regardless of where they occur, this includes skeletal muscle as well as cardiac and smooth muscle, liver, kidney, skin and bone etc. At the tissue/organ level, skeletal muscle consists of over 600 individual named striated skeletal muscles that can be anatomically isolated [9]. These skeletal muscles can vary greatly in length and size, as well as in their muscle fibre composition across the body, i.e., skeletal muscle is not one homogeneous tissue.

### 1.2 Problems Using FFM as a Surrogate for Skeletal Muscle

As muscle contributes the largest portion of FFM, the term FFM is often, incorrectly, used synonymously by many authors to denote skeletal muscle. Two magnetic resonance imaging (MRI) studies of healthy individuals of approximate similar age and BMI estimated that skeletal muscle mass comprised approximately 45%–50% of fat‐free mass [10, 11]. Clearly approximately 50%–55% of fat‐free mass is made up of other components, which include water, bones, and organ tissues. Just as the overall composition of FFM is not static from person to person, and can change with age, sex, race, illness and physical activity, the amount and composition of skeletal muscle is also not static [12]. For example, data from a study of persons less than or equal to 40 years showed that the ratio of MRI‐derived appendicular skeletal muscle to lean soft tissue was inclined to increase with a higher fat mass index (FMI–FM/Ht^2^) up to the third or fourth quintile of FMI, but to decrease in the fifth FMI quintile [13]. This change in ratio was more evident in the upper body, and more so in the arms than the legs.

Fat‐free mass and FM can be highly correlated [14] and are therefore not independent variables in risk modelling. Importantly, increasing body weight driven by increasing adiposity and not by increased physical activity or resistance training, can result in increases in both the FM and the FFM compartments. In these instances, the quality of FFM and especially the quality of skeletal muscle will be negatively impacted as adiposity increases, as will be discussed below. From a diagnostic perspective, the connective tissue in the adipose tissue matrix gets counted as part of FFM inflating the FFM component [10, 15]. Unfortunately, there is no way of knowing the exact contribution of skeletal muscle per se to any gains or losses in FFM in most studied populations that use the 2‐C bc model.

While Lagace et al. [2] suggested that FFM should be indexed to height (FFMI), this does not overcome the contextual problem of concurrent adiposity status. Two persons could have identical FFMI's but vastly different levels of adiposity, not only in totality but also in body location; therefore, health risks cannot be attributed solely to FFMI. Nor does indexing FFM to height squared (i.e., FFMI) necessarily fix the correlation between FFM to FM; Zaniqueli et al. [16] found that after adjusting FFM for height squared, the correlation between FFMI and fat mass (*r* = 0.63) in their cohort was higher than the correlation of absolute FFM and fat mass (*r* = 0.56). Clearly, indexing FFMI to indices that can account for body fat is required to obtain a more accurate correlation.

Lagace et al. [4] also recommended that FFMI should also be adjusted by FMI to “allow for the assessment of FFM per se” [1]. If adjustment means dividing FFMI by FMI, this simply gives the muscle‐to‐fat ratio (MFR), which has been shown in several studies to be negatively associated with insulin resistance or metabolic syndrome [17, 18, 19]. A 10‐year prospective Korean study looking at the conversion of prediabetes back to normal glucose status observed that those with a higher baseline MFR independently had a greater chance of returning to normal glucose status (hazard ratio per standard deviation, 1.15 (95% CI,1.04–1.26) [20]. Other studies have shown that adjusting FFMI or ASMI for FM or FMI does not necessarily give a positive relationship of FFM with metabolic syndrome or insulin resistance [16, 21, 22, 23]. An issue with all studies that use the 2‐C model is that the composition of FFM, and the morphological quality of skeletal muscle could vary between the different study groups giving different results. This may not be a concern in groups such as lean athletes, but it can become increasingly problematic with increasing adiposity and therefore a higher, but unaccounted for, risk of ectopic fat deposition in skeletal muscle.

The limitations of evaluating FFM and FM in isolation saw Prado, Siervo and Wells [24, 25, 26] put forward the concept of load‐capacity, whereby the assessment of health risk by FM and FFM was integrated, rather than determined separately. The principal of load‐capacity is that FM represents metabolic load, and muscle mass represents metabolic capacity [24]. Bosy‐Westphal has also stressed the need for concurrent assessment of lean and fat content in the evaluation of obesity risk [27].

### 1.3 Muscle Morphological Quality

**FIGURE 2:**
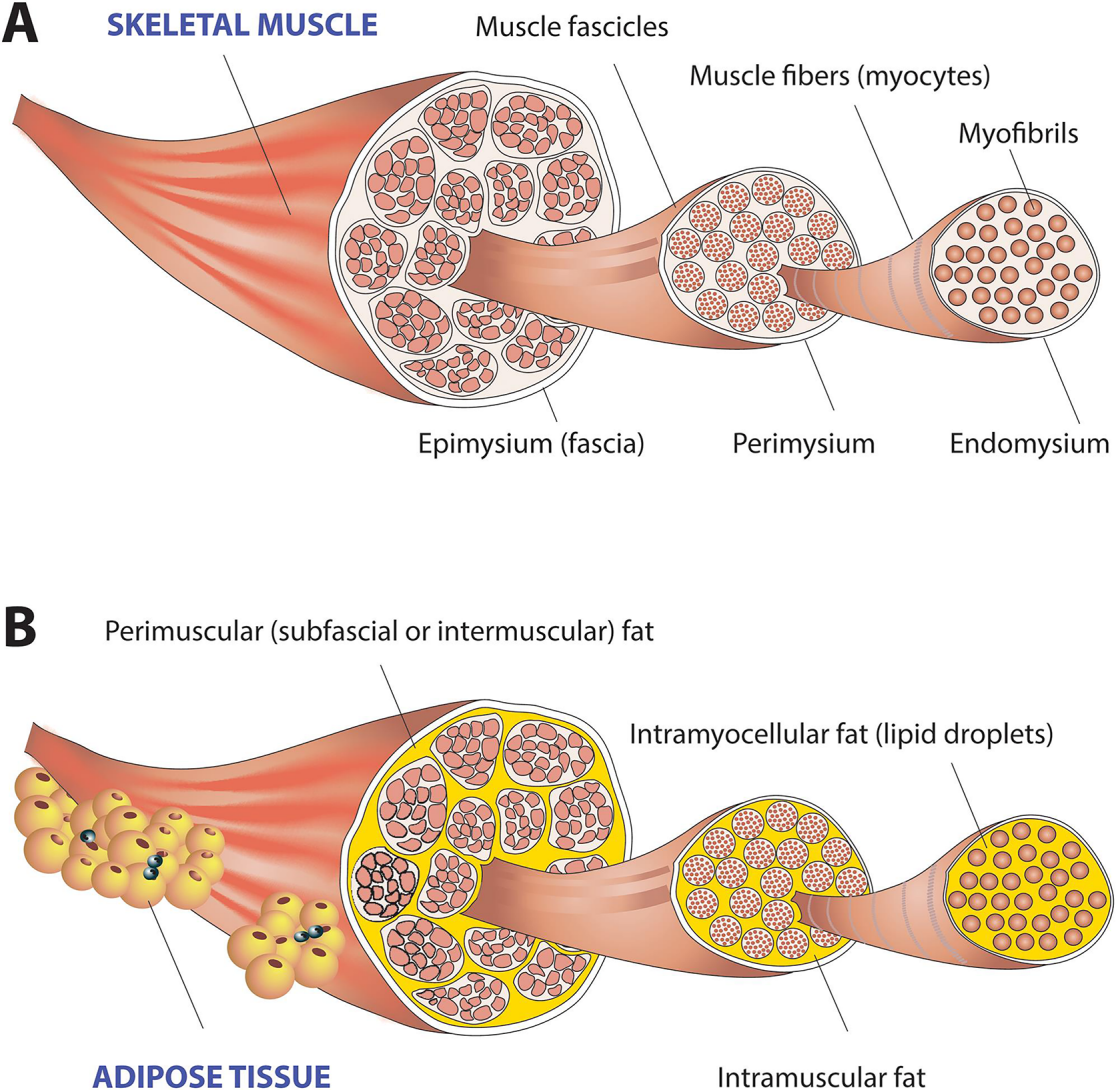
Skeletal muscle and fat deposition. (A) Skeletal muscle is made up of intramyocellular myofibrils, muscle fibres and fascicles bound together by successively thicker connective tissue layers as endomysium, perimysium, and epimysium. (B) Skeletal muscle fat may be classified as intramyocellular (lipid droplets filling the cytoplasm between myofibrils of elongated myocytes) and extramyocellular components. Adipocytes may infiltrate muscle fibres (intramuscular fat), fascicles (intermuscular fat), or exist around the epimysium as extramuscular fat depots of adipose tissue. Reprinted with permission [28].

The morphological quality of skeletal muscle, not just muscle size, is a critical issue with respect to the influence of muscle on health outcomes. With increasing levels of adiposity there is an increase in subcutaneous and visceral fat, the latter significantly related to increased health risk; there is also a higher risk of fat infiltration, ectopic fat, into skeletal muscle, Figure 2.

**FIGURE 3:**
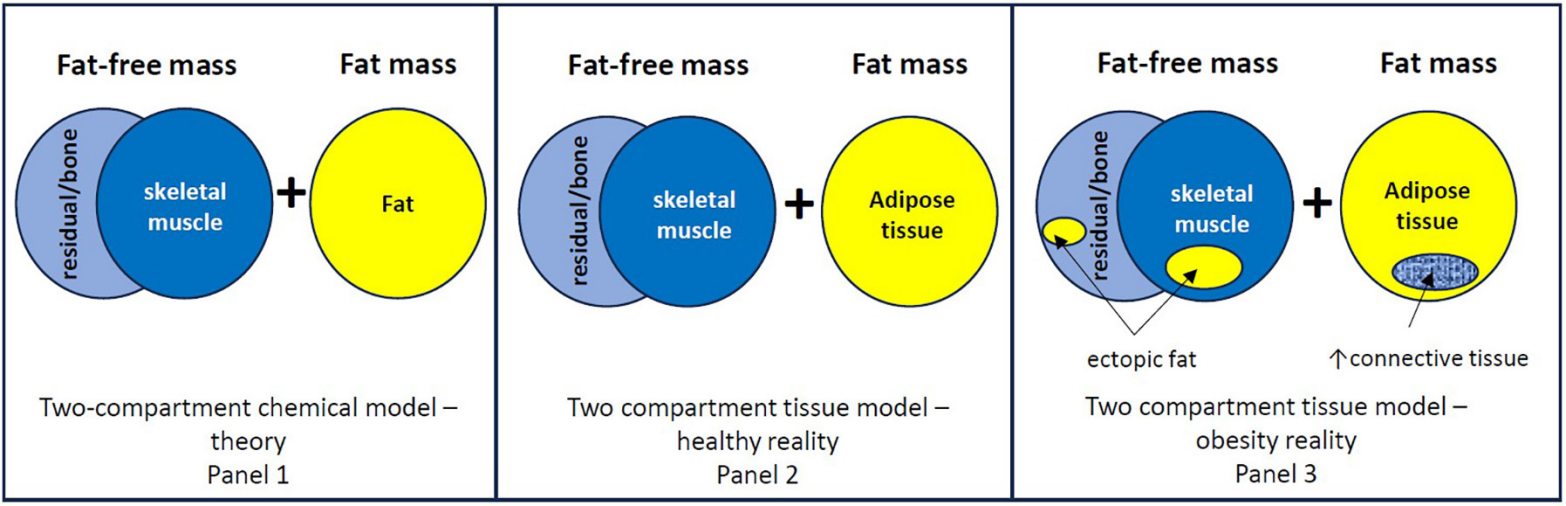
Infiltration of fat into muscle with ectopic fat deposition and increased non‐fat‐free mass connective tissue in adipose tissue with obesity blurs the two‐compartment model at the tissue level and attribution of risk to increased fat‐free mass.

Skeletal muscle ectopic fat can be similar in quantity to abdominal visceral fat and is associated with insulin resistance, type 2 diabetes mellitus, and ageing [29, 30]. Lagace et al. [2] proposed that muscle fat infiltration could be one possible reason for the FFM increasing insulin resistance findings. Critically however, ectopic fat deposition occurs not just in skeletal muscle but in other FFM components such the liver and pancreas, which can also contribute to insulin resistance [31]. The 2‐C bc model equation explicitly states that the two compartments, FM and FFM, are separate and additive (see Figure 3, Panel 1 and Panel 2). Yet, at some point with increasing levels of adiposity, which may vary according to total body mass, age, sex, race and genetics [32, 33], fat and muscle tissue can become interlinked (see Figure 3, Panel 3) but neither ectopic fat nor visceral fat are individually quantifiable by a 2‐C bc model. Consequently, this raises questions about the linking of skeletal muscle to insulin resistance via indirect estimates of FFM. Even when using MRI, the contextual issue of muscle and adiposity needs consideration. As example by Addison et al., two people can have very similar cross‐sectional muscle areas in their thighs as measured by MRI but differing levels of fat infiltration resulting in divergent muscle mobility and function outcomes [34].

The 2‐C bc model estimates of FFM cannot provide an adequate representation of skeletal muscle health in many cases. This is compounded by the fact that 2‐C model also does not provide data on the distribution of adipose tissue across the body, especially important when considering gender risk, nor on visceral fat or on ectopic fat deposition, not just in skeletal muscle, but also in the liver and pancreas. This suggests that risk modelling based on the 2‐C bc model may be inadequate. Lagace et al. [4] using NHANEs data reported that a higher FFMI in men or appendicular FFMI in women was associated with risk factors for metabolic syndrome in both young people (average age ~34 years) and older persons (average age ~64 years). Pearson's correlation coefficients were calculated for FFMI and appendicular skeletal muscle mass (ASMM) with insulin resistance score after adjusting for several confounders including waist circumference, total fat mass and FMI. For younger men, the Pearson *r* value for FFMI was only 0.22, and for older men *r* = 0.12; FFMI for younger women and older women, the coefficients were also low at 0.18 and 0.21, respectively. For ASMM, the results were 0.17 and 0.13 for men, and 0.13 and 0.20 for women. These correlation coefficients were very weak, but statistically significant most likely owing to the sample size, meaning that other factors account substantively more for the risk of metabolic syndrome than either FFMI or ASMM. Although multivariate regression analysis with insulin resistance score as the dependent value was also conducted and while β‐coefficients for appendicular FFMI were statistically significant, no information on the strength of the overall model was given, nor was there any mention of testing for multicollinearity.

### 1.4 Muscle Fibres and Capillarization

Lagace et al. [2] also suggested that an imbalance of type I to type II muscle fibres as well as a reduction in muscle capillarization as possible explanations for decreased insulin sensitivity in persons with higher FFM. While there is an inverse relationship between type I fibres and BMI, and a positive correlation of type IIx fibres and BMI, the positive association of obesity or insulin resistance with type 2 fibres appears to associated with type IIx fibres, not necessarily type IIa fibres [35, 36]. There is complexity at the muscle fibre level, with different rates of atrophy and muscle fibre mix across different muscles, which can greatly complicate study generalizations [37]. For example, with ageing there is an increased risk of insulin resistance and T2D [38] but a decline in the number of type II muscle fibres, as well as a decline in muscle size, strength and power, all which can result in poorer functional outcomes [39, 40, 41]. This is the opposite to the Lagace model of increasing type II fibres and a large FFM as a negative risk factor for metabolic health.

### 1.5 FFM and Biological Plausibility

It was also suggested that “having a larger FFM and larger type II muscle fibres could act as a greater reservoir, thus leading to an increased capacity for ectopic fat accumulation” [2]. As pointed out previously, a larger FFM may often be associated with a larger fat mass, at a certain level, this increased fat mass could infiltrate both the muscle and other organs leading to impaired insulin sensitivity. Disease, obesity, lack of physical activity or exercise, sedentary behaviour and age, all contribute to muscle ectopic fat deposition and the sequence of events leading to insulin resistance in the muscle and organs are often a result of inadequate diet and insufficient physical activity i.e., combinations of sedentary behaviour, insufficient physical activity and inadequate diet (excess energy, unbalanced macronutrients etc.) [42]. It is worth noting that even short‐term (i.e., days) muscle disuse can invoke muscle loss and insulin resistance even without fat infiltration [43]. In persons with a normal BMI, poor body composition as defined as higher‐than‐expected body fat and poorer muscle mass can elicit metabolic disorder. In middle‐aged and older adults, there is the added complication of age‐related muscle mass loss and poorer muscle quality usually accompanied by declining physical activity. There is also the matter of the bidirectional nature of muscle health and chronic disease. Poor muscle health can be a factor in type 2 diabetes risk, but T2D itself can negatively impact skeletal muscle health. Skeletal muscle mass and strength are decreased in T2D. Increasing physical activity, including resistance training, as well as appropriate nutrition will result in less muscle fat infiltration (and other organs) and better muscle capillarization. This will allow better nutrient flows, better muscle health and better insulin sensitivity. Skeletal muscle is not the causal agent of poor metabolism, but the intermediary between lifestyle, ageing factors and metabolism.

### 1.6 Mortality Data Does not Support the Purported Negative Association Between Skeletal Muscle and Health

While fat‐free mass and skeletal muscle size has been implicated in poorer metabolic control, the impact on other critical outcomes such as mortality is still not clear. Sedlmeier et al. [44] investigated body composition data combined from seven prospective studies (KORA, SHIP and NHANES) with a median follow‐up of 14 years; the primary outcome was the effects of FFMI and FMI as measured by BIA on mortality. FFMI and FMI were adjusted for each to isolate the effect of each component (as recommended by Lagace et al. [1]), other variables such as smoking and physical activity were also included. Cox regression analysis showed that FFMI (FFMI 21.9 vs 16.1 kg/Ht^2^, HR 0.7, 95% CI 0.56, 0.87) was protective against total mortality [44]. The opposite conclusion was found for FMI, a higher FMI was associated with increased mortality (FMI 13.0 vs 7.3 kg/Ht^2^, HR 1.52, 95% CI 1.24, 1.86). Sensitivity analysis was conducted by excluding the first four years of follow‐up with no meaningful change in outcomes.

In a UK Biobank report using MRI data from over 39 000 participants, thigh fat‐free muscle and thigh muscle fat infiltration were combined to define adverse muscle composition, i.e., low muscle volume combined with high muscle fat infiltration, and this risk profile was assessed against risk of all‐cause mortality [45]. Those persons with an adverse muscle composition had mortality hazard ratios similar to that found between smoking and cancer [45]. In addition, adverse muscle composition associated mortality occurred in persons with either good or poor physical function (handgrip strength, self‐reported gait speed, falls in previous 12 months), though the latter group fared the worst [45]. Similar results were seen by the same authors using MRI scans to assess UK Biobank participants with non‐alcoholic fatty liver disease [46, 47].

The results of these two prospective study reports highlight that adequate fat‐free mass or skeletal muscle mass is important to overall mortality risk, and that adiposity is also an influential risk factor, and as the UK Biobank MRI study shows, the impact of ectopic fat on muscle is particularly important.

### 1.7 We Need Alternative Measurements to FFM and FM

DXA and BIA are two of the most commonly used estimations of body composition techniques in research and health settings, and estimates of FFM or FM are their most frequently used outcome measures. MRI and CT allow more valid estimates of skeletal muscle volume or mass and in‐depth assessments of muscle and adipose tissue placement, i.e., subcutaneous, visceral and, importantly, within‐muscle fat infiltration [45, 48]. Prospective and cross‐sectional studies have shown the superiority of either CT or MRI compared to DXA in detecting significant changes in skeletal muscle [49, 50, 51, 52, 53]. However, owing to the expense, relative scarcity of the scanners, scan time involvement and potential radiation exposure in the case of CT, MRI and CT have historically been used in body composition studies with relatively small sample sizes. Advancements in MRI and CT technology and data processing allow for much faster scan and processing times, permitting much larger study sample sizes. In ongoing extensive studies, MRI body composition data linked to individuals' health data will provide important body composition data insights into health outcomes [54, 55].

In a study of UK Biobank MRI follow‐up data gathered over two years in over 3000 persons, significant changes in MRI‐measured body composition and muscle function were observed, despite no overall substantial changes in body mass index (BMI), body mass or waist circumference [56]. Small but significant declines in handgrip strength were linked to small decreases in various MRI skeletal muscle measurements. Increases in visceral adipose tissue and thigh intermuscular fat were observed despite no changes in BMI, waist circumference or overall ectopic‐fat deposition [56]. This study highlights the ability of MRI to detect small, clinically meaningful body composition changes even within a relatively short timeframe.

Nevertheless, MRI and CT cannot be reasonably deployed in individual healthcare settings, requiring other methods for potential clinical use. D3‐creatine (D_3_Cr) assessment is an emerging method of whole‐body skeletal muscle estimation. Several studies show a good correlation between D_3_Cr with MRI muscle volume; however, large differences in limits of agreement exist upon Bland Altman analysis [57]. Current limitations of D_3_Cr are the valid estimation of the creatine pool size across healthy and clinical populations owing to several unresolved issues. These are—totality of tracer retention, confinement of tracer to the skeletal muscle, potential creatine concentrations differences between different skeletal muscles, and the effect of factors such as gender, diet, creatine supplementation, exercise, pregnancy and age [58]. Broader limitations of D_3_Cr are that it cannot provide regional skeletal muscle or ectopic fat assessment nor be used “on‐demand” in everyday clinical assessment.

Bioelectrical impedance analysis (BIA) would be the most widely used body composition assessment method outside of simple anthropometry. In addition to estimating FFM and FM, BIA can provide data outputs such as estimates of extra‐, intracellular, and total body water, as well as phase angle (PhA); notably, PhA is not dependent on external validation equations as are FFM and FM. These additional outputs, particularly PhA, can provide helpful information about muscle health and health risk [59, 60].

The issue of muscle ectopic fat remains a critical issue to all techniques for the reasons previously stated. Addison et al. [34] provided an example of two individuals with similar thigh muscle areas measured by CT but differing amounts of ectopic fat, resulting in different functional outcomes between them. Therefore, even CT or MRI estimates of muscle need contextualizing with regard to ectopic fat [61]. Similarly, even if DXA, D_3_Cr, or BIA estimates of FFM or muscle show a good correlation with MRI or CT estimates of muscle mass, this may be inadequate if muscle quality (i.e., ectopic fat) is not also measured. BIA offers this possibility with the use of PhA. A Japanese study of older adults found leg PhA measured by segmental BIA correlated with CT‐derived thigh mean attenuation value (a measure of ectopic fat), mid‐thigh muscle‐cross‐sectional area, and skeletal muscle index [62]. A study of breast cancer survivors found correlations between PhA and cardiorespiratory fitness, thigh skeletal muscle volume, and thigh myosteatosis measured by MRI [63]. Unfortunately, neither study measured muscle function, however PhA has demonstrated a correlation with handgrip strength and physical function [64, 65, 66]. Therefore, PhA can correlate not only with skeletal muscle and indices of muscle fat infiltration, but also with muscle strength and physical function, offering potential health risk insights beyond that of FFM.

PhA data can provide further insights by scaling resistance and reactance outputs by height, i.e., bioimpedance vector analysis method (BIVA), or by scaling by height and cross‐sectional area, i.e., specific BIVA [67]. All PhA indices require population‐specific normative data, however, there is a lack of standardization of raw data outputs between manufacturers' BIA machines limiting the generalizability between different study outcomes and existing normative data [68]. Hopefully, these issues can be addressed through the newly formed BIA International Database [69]. The problems of generalizability and device‐dependent normative data also apply to FFM and FM from BIA and DXA.

The future of body composition will likely involve its integration with muscle function and CRF assessment, leveraging insights from omic‐based platforms [70] and using artificial intelligence, including machine learning [71]. Advances in devices, for example, BIA on a watch [72] and 3D anthropometric body composition assessment through mobile phones [73] also offer new patient‐centric and remote data collection opportunities. Body composition assessment and its role in healthcare have great potential and will likely look much different from how it is utilized today.

### 1.8 Resistance Training and Health Outcomes

Following the idea that FFM potentially negatively affects metabolic health, exercise recommendations to maintain or enhance skeletal muscle mass were also queried [1, 2, 7]. While Paquin et al. [7] confirm “RT (resistance training) is a highly relevant and proven strategy to prevent T2D” they question “if the current focus on SM (skeletal muscle) hypertrophy, or the prevention of SM losses during weight loss trials is appropriate amongst all populations.” There are several factors that need consideration with this concern.

Firstly, as discussed, it is essential to recall that FFM at the molecular level of body composition assessment is not synonymous with skeletal muscle at the organ/tissue level of assessment and measurement of FFM may not adequately reflect what is occurring to skeletal muscle. An example of this confusion is in commentary by Lagace et al. [2], discussing the results of a meta‐analysis [74] which concluded that while resistance training was useful in improving HbA1C, this effect was independent of changes in FFM; Lagace et al. suggested this means that mechanisms other than those related to FFM could be at play. One of these other potential mechanisms could be the benefits of the resistance training in decreasing muscle and hepatic ectopic fat content separate to any change observable in FFM measurement. In the STRONG‐D trial of persons with a normal‐weight T2D, strength training showed the best effect on HbAc1 reduction compared to aerobic training or combined strength and aerobic training [75]. The best predictor of a HbA1C reduction was an increase in lean mass as well as a reduction in fat mass, The authors of this paper contrasted their results to other trials in overweight or obese T2Ds, where strength training was less effective than combined training [76, 77]; they concluded that reduction in fat mass may be more of a priority in these settings. These results underscore the contextual nature of body composition assessment.

Secondly, an important fundamental question is what constitutes hypertrophy. How much muscle gain is physiologically detrimental, and under what conditions? For example, there is a phenotype where individuals may have a normal body mass index but carry high levels of body fat [78]. Similarly with ageing, persons may lose skeletal muscle, gain body fat, with no net change in body weight. In these cases, would ‘hypertrophic’ resistance training, which helped restore normal body composition with no net change in body weight be considered bad? Is preventive resistance training that offsets age‐related declines in muscle mass with hypertrophic resistance training bad? We think not.

Thirdly, an important consideration is the artificial demarcation between hypertrophic or strength training. While there are training protocols that favour strength or hypertrophy outcomes, the outcomes are not binary, i.e., one does not have only muscle mass gains or only muscle strength gains. Strength training can elicit gains in both in strength and fat‐free mass [79, 80, 81] and hypertrophic training can not only increase muscle size but also muscle strength. A systematic review comparing hypertrophic resistance training to endurance resistance training in T2D concluded that either training regime was likely to be beneficial [82]. For most people hypertrophic resistance training is self‐limiting i.e., continued resistance training does not produce unlimited muscle gains. By contrast, very high levels of hypertrophic resistance training for bodybuilding that produce very large muscle sizes, are not synonymous with resistance training prescriptions for general good health, and the high degrees of muscularity achieved with such protocols are partially attributable to extreme dietary practices and the potential use of steroids and performance‐enhancing drugs in some groups.

Epidemiological studies show a benefit in persons undertaking resistance training regarding non‐communicable diseases, including T2D [83, 84, 85]. In a study of NHANES data, researchers compared self‐reported resistance training activity to HOMA‐IR levels in 6561 non‐diabetic persons [86]. After adjusting for several potential confounding variables including waist circumference, fat mass, percentage body fat and FFMI, the odds ratio of insulin resistance in men who did no strength training compared to those who undertook moderate to high levels of resistance training was 1.58; no effect was seen in women.

Both resistance training and endurance training can have positive effects on muscle health including decreasing muscle fat infiltration [87]. Intervention studies show a wide range of benefits for resistance training alone, or better still, when combined with aerobic training in T2DM [88, 89]. Resistance training, if done appropriately, can increase cardiorespiratory fitness (CRF) [90, 91, 92, 93], while endurance training may increase skeletal muscle health [94, 95]. The potential effect of resistance training on CRF is potentially very important given that the role of CRF in mortality risk [96]. Finally, while resistance training specifically targets skeletal muscle, there are multiple non‐muscle benefits to the body; readers are referred to the paper of Sawan et al. [97] for more details.

Undoubtedly, changes in body composition and health outcomes in resistance training may result not just from the training itself, but also from possible dietary changes and other health halo effects that can occur with any form of exercise initiation. The outcomes of resistance training will also depend on adequate protein and energy, vitamin D, omega‐3 fatty acids etc.). Adequate controlling for these factors is another potentially important confounding factor in exercise trials, as are other physiological measures such as CRF; all are crucial in contextualizing overall body composition and fitness and evaluating morbidity and mortality risk.

Nowadays, there is recognition that overall muscle quality or muscle health rather than just muscle size per se as being important. This is seen with the pivot from defining sarcopenia simply as a deficit of muscle mass to one emphasizing muscle strength as a diagnostic factor and rehabilitation goal [98]. Muscle strength and muscle power are critical as these can be affected by age and disease to a greater extent than muscle mass, and both often are more strongly associated with positive health outcomes than muscle mass per se; they also respond well to resistance training, regardless of muscle hypertrophy [99, 100, 101, 102]. However, to be clear, the differences observed may have been owing to the body composition methods used, and sufficient muscle mass is critical to overall health, and its diminution with age and disease means that skeletal muscle may need to be replaced or maintained, therefore requiring muscle‐building exercise to compensate for these losses. Given the large demographic shift in many countries in the increased prevalence of older people, resistance training of all sorts will become increasingly important alongside proper nutrition.

Furthermore, resistance training for health is not about superfluous hypertrophy; we are unaware of any health guidelines expressing the opinion that more is always better regarding muscle mass. Resistance training can be undertaken at any age, from children and teenagers [103] up to nonagenarians [104], with positive health benefits. The effectiveness of resistance training is why the American Heart Association [105], the American Diabetes Association [106], and various other health bodies, including the World Health Organization [107], recommend it as part of a regular physical activity/exercise program.

## 2 Conclusion

The proposal that higher FFM is negatively associated with metabolic health and consequently, that hypertrophic resistance training, a form of training that increases skeletal muscle, a component of FFM, is problematic. A major issue is the synonymous use of the ‘fat‐free mass’ to denote skeletal muscle mass. The suggested causes of how skeletal muscle via FFM may be implicated in metabolic health, also help explain why the 2‐C bc model is not necessarily adequate for the task. Fat and fat‐free mass are correlated and often increase together with increasing adiposity, i.e., a high FFM (indexed or not), can co‐exist with high levels of body fat. The 2‐C bc model outcomes of fat and fat‐free mass cannot account for muscle morphological quality, nor for the distribution of adipose tissue. The coexistence of a high FFM and FM are most likely to occur because of lifestyle choices such as inadequate physical activity and exercise along with poor dietary choices. These same factors that negatively impact muscle health can also negatively impact other non‐skeletal muscle components of FFM such as the liver and the pancreas which also can affect metabolic health. The ‘counterintuitive’ findings of a positive relationship between skeletal muscle assessed by the 2‐C bc model and poor metabolic health, may be more of an artefact than a causal relationship. Clearly, the area of body composition and disease risk is complex; the work of Lagace et al. has highlighted the problems with the most popular current body composition assessment techniques, but also in the interpretation of the data outcomes. Rather than just urging care when reporting FFM, surely it is time to move beyond FFM to embrace more direct measures of skeletal muscle and its morphological and functional qualities; a more granular examination of skeletal muscle will allow better individual assessment for disease risk estimation. Fat mass also needs better reporting regarding its distribution and if possible visceral and ectopic depots. Both components need assessing in relation to age, sex, race, and in context to each other. To fully understand the outcomes of studies that incorporate body composition findings, the use of techniques, such as MRI or CT, which can provide better clinical and mechanistic insights are preferred. Advancements in technology mean that body composition scanning with MRI may be performed far more rapidly than previously, allowing for involvement in larger projects or trials, as exampled by the UK Biobank project. In the meantime, currently available technology such as BIA that provide enhanced or non‐2‐C bc model assessments, can possibly provide more useful insights into muscle quality and clinical outcomes than 2‐C bc model outcomes alone. The premise that a higher skeletal muscle/FFM can impair metabolic health, again may be more a result of the inadequacy of the 2‐C bc model as well as complexities with resistance training and trial designs, rather than with skeletal muscle itself. The assertion that hypertrophic training is bad while strength training is good, is undercut by the fact that it is possible to have muscle mass and strength gains with both forms of training i.e., there is not exclusive binary outcomes with different training types. Muscle hypertrophy is not the primary outcome of resistance training required for metabolic health, and for most people hypertrophic resistance training is self‐limiting anyway. This paper contends that the evidence against resistance training is likely confounded by the measurement techniques (2‐C bc model); and that the evidence for resistance training, either by itself or with aerobic training, should encourage it is use as part of a lifestyle strategy to maintain good health and decrease the risk of chronic disease across the lifespan.

## Conflicts of Interest

The authors declare no conflicts of interest.
